# Factors determining the occurrence of anthropogenic materials in nests of the white stork *Ciconia ciconia*

**DOI:** 10.1007/s11356-018-1626-x

**Published:** 2018-03-13

**Authors:** Zuzanna A. Jagiello, Łukasz Dylewski, Dominika Winiarska, Katarzyna M. Zolnierowicz, Marcin Tobolka

**Affiliations:** 1 Institute of Zoology, Poznań University of Life Sciences, Wojska Polskiego 71C, 60-625 Poznań, Poland; 2 Department of Zoology and Physical Anthropology, Complutense University of Madrid, Jose Antonio Novais, 12, 28040 Madrid, Spain

**Keywords:** Nest-building behaviour, Breeding success, Debris, Pollution

## Abstract

Birds have been using anthropogenic materials for nest construction for the past few decades. However, there is a trade-off between the use of new nesting material, which is often linked to greater breeding success, and the higher risk of nestling mortality due to entanglement or ingestion of debris. Here, we investigate the incorporation of anthropogenic materials into nests of the white stork *Ciconia ciconia*, based on a long-term study of a population in Western Poland. We recorded at least one item of debris in 50 and 42% of nests at the egg and nestling stages, respectively. More debris was found in nests located in territories with higher number of anthropogenic material in the surrounding environment. We found a relationship between the age of females, the number of debris in the area surrounding a nest, and the number of debris in the nest. We found no significant effect of the total number of debris in nests on clutch size, number of fledglings, or breeding success. Studies on the influence of the age and sex of individuals in understanding this behaviour and its drivers in bird populations should be continued.

**Electronic supplementary material:**

The online version of this article (10.1007/s11356-018-1626-x) contains supplementary material, which is available to authorized users.

## Introduction

Human activities induce significant changes in the natural environment, which lead in turn to changes in the behaviour of animals living in urban environments (Carney and Sydeman 1999; Slabbekoorn and den Boer-Visser 2006; Miranda 2017). Increases in solid waste abundance as a result of the growth of urbanisation (Hoornweg et al. 2013) have made anthropogenic materials commonly available in terrestrial and marine environments. Debris, mainly in the marine environment, is a cause of mortality in many animals (Gregory 2009; Ryan et al. 2009; Votier et al. 2011). However, it is also used as material for the construction or padding of nests. Birds of different taxa are well known for incorporating anthropogenic materials into nests (Morin and Conant 1990; Huin and Croxall 1996; Blem et al. 2002; Hartwig et al. 2007; Townsend and Barker 2014). This behaviour may be influenced by the abundance and availability of debris in marine, urban, and agricultural environments (Henriksen 2000; Wang et al. 2009; Bond et al. 2012, 2013; Eriksen et al. 2014; Wilcox et al. 2015). Due to environmental changes (e.g. large-scale modern farming, the development, or spread of urbanisation), natural elements (e.g. wooden sticks, straw, hay) may be scarce. Hence, incorporating easily available debris can potentially reduce the costs of collecting natural material, especially when the debris is light and durable, e.g. plastic string (Antczak et al. 2010) or plastic foil. A study of the black-faced spoonbill *Platalea minor* showed that, in a highly polluted and changed environment, supplying natural elements led to a reduction in the number of debris incorporated into nests (Lee et al. 2015). Collection of debris may be modified by several other factors. In terrestrial species, the number of anthropogenic materials used to construct the nest can be correlated with the level of urbanisation (Wang et al. 2009; Radhamany et al. 2016). However, the use of anthropogenic materials in nests may also be triggered by mating behaviour, e.g. bowerbirds (Ptilonorhynchidae) build bowers to attract females (Borgia 1985). Bower decoration can be a decisive factor for females choosing a mate. Bowerbirds decorate bowers with flowers, plants, and debris—bottle tops, straws, etc. (Borgia 1985). Males with better decorated bowers are more attractive and have better chances for reproduction; this may ultimately increase the number of anthropogenic material in bowers. Incorporation of debris may be also dependent on the age (experience) of individual birds (Coleman et al. 2004). In black kites *Milvus migrans*, the number of anthropogenic material in nests is related to the age of pair members; it is also strongly linked to individual quality and therefore has a strong influence on breeding success. Higher-quality individuals collect more debris, are better breeders, and occupy better territories (Sergio et al. 2011). What is more, nest decoration can signal the condition, experience, fighting capabilities, territory quality, and social dominance of the individual or the breeding pair to other individuals (Canal et al. 2016).

The use of anthropogenic materials may also have negative consequences. Plastic string, fishing nets, and angling gear are resistant materials occurring in the sea (far from nest sites) as well in the nest (collected by adults). These items often cause entanglement, leading to mortality or injuries (Baker et al. 2002; Seacor et al. 2014). Entanglement has been recorded in 25% of 312 studied seabird species (Gall and Thompson 2015). Hence, birds collecting debris for their nests may increase the risk of ingestion and entanglement, which may reduce breeding success (Mee et al. 2007). While this phenomenon has been studied in marine birds (Provencher et al. 2017), it is rarely described in the cases of land birds and inland waterbirds. Other debris, e.g. cigarette butts, may have an initial positive effect, i.e. may act as an ectoparasite repellent (Suárez-Rodríguez et al. 2012). However, long-term observations showed the cost of exposure to toxins, namely, a higher genotoxicity level (damage to DNA or chromosomal material) in nestlings’ blood cells (Suárez-Rodríguez and Macías Garcia 2014). Ingestion of anthropogenic material, which may also occur in nests, is another cost associated with individual survival (Houston et al. 2007; Mee et al. 2007; Young et al. 2009; Henry et al. 2011; Finkelstein et al. 2012).

In this study, we examine the impact of the incorporation of anthropogenic materials into nests of a large migratory synanthropic water bird, the white stork *Ciconia ciconia*. Traditionally, this bird bred in colonies in river valleys but has now moved closer to human settlements. Nowadays, it is a common species which breeds and forages in agricultural areas and nests close to human settlements (Schulz 1998). At the same time, it is a well-studied bird species, with populations being monitored over the long term (Bairlein 1991). The population has declined in Western Europe due to the intensification of agriculture and to drought in the Sahel, where it winters. Following a precipitate decline, the population has recovered due to reintroduction and additional feeding programmes in several countries (Bairlein 1991). In Western Europe, white storks often forage in rubbish dumps, a practice which has probably driven the population increase (Tortosa et al. 2002). This species is known to incorporate debris from rubbish dumps in their nests (Henry et al. 2011). Eastern (including Polish) white stork populations rarely use rubbish dumps (Kruszyk and Ciach 2010); instead, debris from the immediate environment (e.g. plastic strings, foil) is collected from agricultural lands as nesting material and incorporated into nests (Tryjanowski et al. 2006). Plastic string is one of the most common anthropogenic materials used by terrestrial species as nesting material (Antczak et al. 2010; Seacor et al. 2014). This material has been commonly used in Polish agriculture since 1982 for tying, e.g. hay and straw (Ptaszyk 1994). Due to its utility, fragments are ubiquitous in the agricultural landscape. It is available to foraging birds, along with plastic foil, which is used in farmlands to cover, e.g. hay bales or certain types of crops. These materials are the main sources of plastic pollution in farmlands. Moreover, the white stork is characterised by its longevity, and thus studies on the impact of age on nesting behaviour are feasible. This makes the white stork a suitable subject for detailed research on the impact of anthropogenic material on nesting behaviour. We investigated two factors which may influence the inclusion of debris in nests: the availability of debris in the vicinity of nests (Henriksen 2000; Wang et al. 2009; Bond et al. 2012) and the age of individuals (Sergio et al. 2011; Canal et al. 2016). With regard to age-assortative mating in white storks, we know that the ages of both breeders constituting a pair are very similar (in cases where the age of only one pair member is known) (Barbraud and Barbraud 1999). Hence, we hypothesised that (1) the greater the density of anthropogenic materials in the environment in the vicinity of the nest, the higher the number of anthropogenic materials incorporated into the nest, associated with the question of whether there is any preference for a particular type of debris; (2) the ages of individuals have a direct impact on the number of anthropogenic material included in the nest; and (3) birds incorporating the greatest number of debris (pieces) into nests are not only older but also better breeders, as reflected in greater numbers of eggs and fledglings. The incorporation of debris into nests in agricultural landscapes has been shown in only a few studies (e.g. Antczak et al. 2010; Townsend and Barker 2014). This is the first long-term research to explain the effect of debris on a bird inhabiting farmlands, i.e. the white stork.

## Materials and methods

### Fieldwork

We conducted the study in Western Poland near the town of Leszno (51°51′ N, 16°35′ E), within a mainly rural area of 4154 km^2^ comprising arable fields (54%), forests (17%), human settlements (10%), a small proportion of meadows (7%) and pastures (< 1%), and other land-use types (12%) (Tobolka et al. 2015).

First, we used data collected during a long-term study of the white stork’s breeding and population ecology, which comprised ca 50 nests visited each year between 2009 and 2016 for recording clutch size, over 100 nests where the number of fledglings was recorded during ringing, and over 300 nests where breeding results were recorded (for details, see Tobolka et al. 2013, 2015). The data were collected for 342 broods at the egg stage and 445 broods at the chick stage during the years 2009–16; during this period, all anthropogenic materials in the nests were counted (details in supplementary material, Table S1). To decrease the risk of nestling entanglement and avoid accumulation of recorded debris during subsequent visits, we removed all debris from the nest during each visit. Second, we searched for adults of known age and sex with alphanumeric rings (sexed by molecular procedures; see details in Dubiec and Zagalska-Neubauer 2006; Fernandes et al. 2006).White storks are marked mainly as nestlings; hence, we knew the exact age of adults that had been ringed several years earlier and re-recorded during this study. Marked breeders of known age and sex can help to explain the possible impact of age and sex on debris-collecting behaviour and parental care. Additionally, we conducted more complex field research during the 2015 breeding season. We conducted two visits: during egg-laying (32 broods) and chick-rearing periods, the latter amounting to 43 broods between 25 and 45 days of age. We visited accessible nests (the same included in the long-term study) with a 7-m ladder, cherry-picker, and climbing equipment. In the course of nest visits, we collected data on clutch size and number of nestlings, and recorded all anthropogenic materials present in nests. Clutch size was recorded during the second half of incubation (between the 15th and 30th days) (details in Tobolka et al. 2015). The number of nestlings was recorded at the time nestlings were marked. We established a buffer area with a radius of 500 m around a nest, in which we created four random transects 150 m long. The width of each transect was 2 m on either side, or 4 m in aggregate, at the beginning of the breeding season (egg stage), and 1 m on either side (2 m in aggregate) during the nestling stage due to the lower level of visibility caused by vegetation growth; however, dimensions were the same for each transect. The foraging range of the white stork is characterised by a radius up to 2 km (Ożgo and Bogucki 1999); however, the stork collects nesting material in the immediate vicinity of the nest, accordingly to personal observations (Tobółka, unpublished). We counted all available potential anthropogenic nest materials lying on the ground (Fig. S1). We recorded only anthropogenic materials with dimensions over 1 cm in diameter and easily detectable by human eyes (e.g. plastic string, foil, paper and other material). Later, we divided debris from white stork nests and transects, according to physical properties and to Townsend and Barker (2014), into categories: foil, plastic string, other plastic, paper, textiles and other. Additionally, we divided nest debris using a standardised method (Provencher et al. 2017) in order to facilitate comparison with future studies. Finally, we recorded breeding success by counting fledglings, i.e. chicks over 50 days of age standing in the nest and considered able to fly (a standard method to estimate the breeding success of the white stork) (Tryjanowski et al. 2006). Breeding success was defined as the number of fledglings divided by the number of eggs laid.

### Statistical analyses

Prior to statistical analyses, we used the fitdistrplus package (Delignette-Muller and Dutang 2015) to check the distribution of dependent variables. To test the effect of the number of debris in the environment on the number of debris in white stork nests, we used a generalised linear mixed model (GLMM) with a Poisson error structure and a log link function; specifically, we used a GLMM with a binomial error structure with a logit link function to explain the effect of age and sex on the presence of debris in nests. As a binomial response variable, we compared nests with debris (1) to nests without debris (0). We used GLMM to model variations in clutch size, numbers of chicks, and breeding success. For each dependent variable, we used a GLMM with a Gaussian error structure and an identity link function with one fixed-effect predictor: total number of debris. In these GLMMs, we used nest identity and year as random factors. To test the white stork’s preference for particular types of debris (plastic string, foil, paper or other), we used chi-square contingency independence tests comparing the percentage of debris between white stork nests and the environment for two stages (egg and chick). We analysed separately the number of debris during the egg and nestling stages because the availability of anthropogenic material might change due to growth of vegetation and intensification of agricultural works (e.g. hay collecting and harvesting) during the breeding season proceeding. All analyses were performed in R, version 3.3.2 (R Development Core Team 2016), using the lme4 (Bates et al. 2015) and ggplot2 (Wickham 2009) packages.

## Results

In the course of the study (2009–16), in 171 of 342 (50%) broods during egg stage and in 186 of 445 (42%) broods during chick stage, anthropogenic materials were present. The anthropogenic materials categories occurred in the following proportion: plastic string (38%), foil (33%), textile (8%), paper (5%), other plastic (7%) and other (9%). In the surrounding area, the proportion of available debris was as follows: plastic string (83%), foil (8%), textile (0.3%), paper (2%), other plastic (3%) and other (3%). The white stork at the chick stage revealed a positive preference for plastic foil (chi-square = 5.828, *p* = 0.02) and a negative preference regarding string (chi-square = 24.858, *p* < 0.001). However, during the egg stage, these relationships were not significant (Table 1).

**Table 1:** Results of a chi-square contingency independence test for white stork preferences for debris type

	Nest (%)	Environment (%)	Chi-square	*P*
Egg stage				
String	55	76	3.366	0.07
Foil	10	13	0.391	0.53
Paper	0	2	–	–
Other	35	9	15.364	< 0.001
Chick stage				
String	30	83	24.858	< 0.001
Foil	21	8	5.828	0.02
Paper	11	2	6.231	0.01
Other	38	7	21.356	< 0.001

**Fig. 1:**
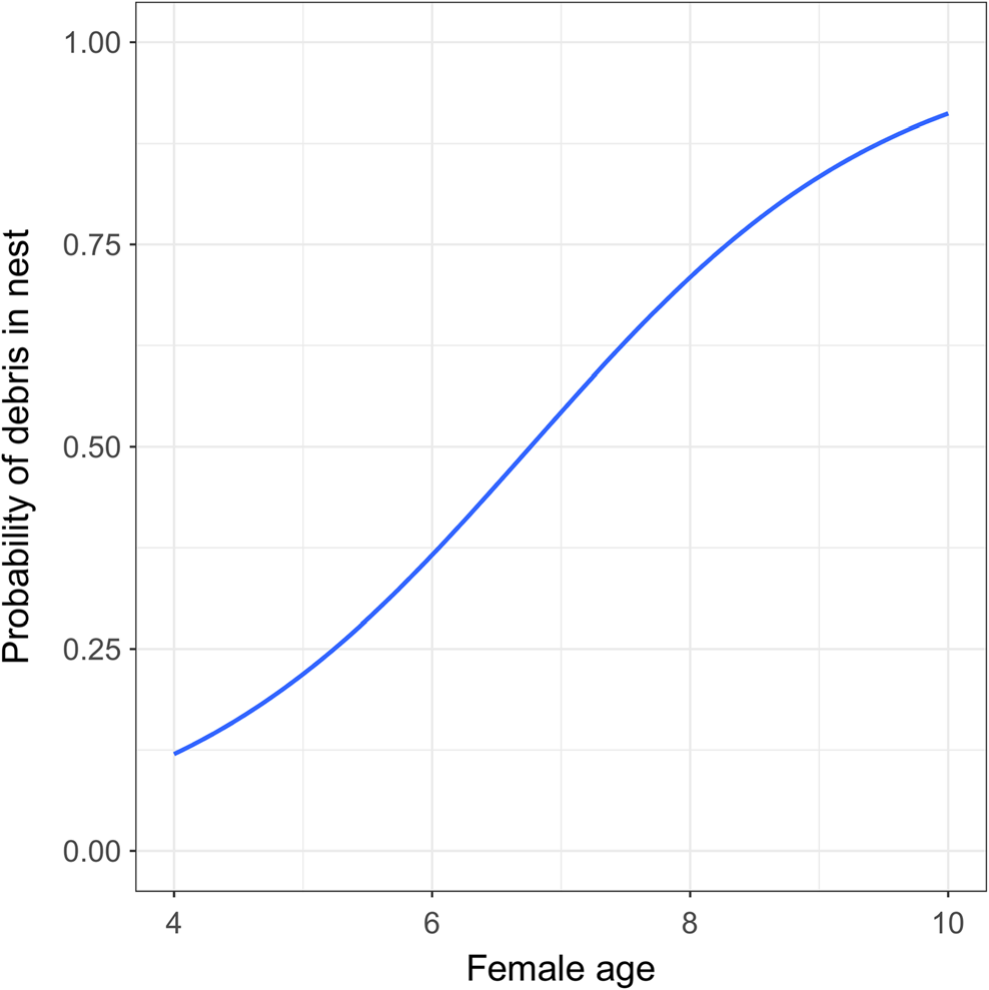
The probability of the presence of debris in nests in relation to the ages of white stork females

More debris was found in nests located in territories with higher rates of anthropogenic material in the surrounding environment (*β* = 0.02075 ± 0.01351, *Z* = 2.006, *p* = 0.0453, *N* = 75).We found that probability of recording debris in a given nest was positively correlated with the age of the female (*β* = 0.9147 ± 0.385, *Z* = 2.377, *p* = 0.018, *N* = 33, Fig. 1). The age of the male did not explain the probability of recording debris in the nest (*β* = − 0.2638 ± 0.320, *Z* = − 0.824, *p* = 0.410, *N* = 20).

We found no significant effect of the total number of debris in a given nest on clutch size (*p* = 0.423, Table 2), number of fledglings (*p* = 0.956), or breeding success (*p* = 0.106).

**Table 2:** The GLMM’s with Gaussian error describing the relationship between clutch size (*n* = 342 broods), number of fledglings (*n* = 445 broods) and breeding success (*n* = 196 broods) to total number of debris in nest

Effect	Estimate	Error	*t*	*P*
Clutch size				
No debris in nest	0.513	0.191	0.802	0.423
No of fledglings				
No debris in nest	4.168e-03	7.499e-02	0.056	0.956
Breeding success				
No debris in nest	0.031	0.019	1.624	0.106

## Discussion

In this study, we showed that 46% of white stork nests contained anthropogenic material. The relationship between the numbers of debris in the vicinity of a nest and in the nest itself was significant. Thus, the white stork, as well as marine birds, may be a potential indicator of debris pollution in the surrounding environment, as incorporation of debris in nests may be related to its availability in the environment around those nests (Votier et al. 2011; Avery-Gomm et al. 2012; Bond et al. 2012). In many aspects of life, the white stork demonstrates its opportunism and ability to adapt to changing environments, e.g. its exploitation of a wide range of new food resources (Tortosa et al. 2002; Djerdali et al. 2008, 2016; Ciach and Kruszyk 2010; Gilbert et al. 2016), its use of new nesting sites, and its tendency to nest close to human settlements (Tryjanowski et al. 2009; Flack et al. 2016); the use of debris as a lining material is another example. The most probable reason for incorporating anthropogenic materials into the nest structure is that these materials are common and easily accessible in the agricultural landscape, which we have shown in the case of the white stork. At the moment, we do not know why white storks prefer to collect foil and plastic, but the preference was shown only during chick stage. During egg stage, there was no preference, and probably white storks randomly use what is available in the local environment. Maybe the former are better insulation materials; however, more detailed studies, including experiments, are needed to confirm this statement. Additionally, older individuals collect these materials to a greater extent in the vicinity of the nest. White storks may select debris in relation to its abundance, as the amount of plastic in the landscape increases (Thompson et al. 2009). Given that natural materials are probably limited in an intensive agricultural landscape (Antczak et al. 2010), the use of anthropogenic nest material may be a beneficial resource in nest construction. Our research showed that the most common items of debris in the immediate vicinity of white stork nests that were incorporated in nests were plastic string and foil. These anthropogenic materials were also most common in nests of the American crow *Corvus brachyrhynchos* (Townsend and Barker 2014) and the great grey shrike (*Lanius excubitor*) (Antczak et al. 2010) in agricultural landscapes.

In our study, the probability of the presence of debris in nests was associated with the age of females. However, we did not find a relationship between the probability of the presence of debris in the nest and the age of male white storks. In this species, both partners collect nesting material, but we do not know whether bringing debris into the nest is a sex-dependent activity. One local study suggests that males deliver more nesting material, particularly in the beginning of the breeding season (Bocheński and Jerzak 2006); however, we have no knowledge of a more general pattern. In regard to age-assortative mating in this species (Barbraud and Barbraud 1999; Ferrer and Penteriani 2003), we can assume that the ages of white stork males were similar to those of their female partners. The lack of a significant relationship may be a result of the small sample size of males (*N* = 20) of known age. Mate choice in the white stork is mostly dependent on nest site occupancy. White storks prefer breeding sites with large nests or a nest that has been occupied continuously for at least one successful breeding season (Bocheński and Jerzak 2006; Vergara et al. 2010). Storks collect and deliver debris to the middle part of the nest as a lining, whereas we recorded only debris in upper parts, which reflects collecting behaviour during the current breeding season; therefore, the age and size of a nest should not influence the number of debris recorded therein within a single breeding season. Some bird studies have shown a relationship between anthropogenic materials incorporated into nests by mates and pair formation (Coleman et al. 2004; Sergio et al. 2011). In this study, numbers of debris were positively correlated only with the ages of females. Sergio et al. (2011) showed that numbers of debris in nests of black kites were greatest for birds aged 8–11 years, whereas in younger and older individuals, this phenomenon was significantly less frequent. The probable explanation is that the experience (better quality) of the individual is age-related, although possibly, it exhibits an inverted U-pattern (Ortega et al. 2017). The white stork also reveals this pattern, i.e. individuals aged between 8 and 12 years are the best breeders (Profus 2006). In our study, the relationship had a different character, being non-linear as well; nevertheless, the probability of incorporating debris into the nest increased continuously with a stork’s age. Experience comes with age, therefore, more experienced birds may be more likely to incorporate debris into their nests, according to results published by Sergio et al. (2011). Irrespective of this, the oldest female in our study was 10 years old, whereas the white stork can live much longer, even up to 39 years of age (Schulz 1998). Therefore, records in subsequent years may provide additional data which will render the character of the relationship more similar to that observed by Sergio et al. (2011).

We found no significant relationships between total number of debris collected for nests and clutch size, number of fledglings, or final breeding success. Assuming that the number of debris collected for the nest is a proxy for experience, we may have observed its effect only on breeding success. Egg counts alone do not explain the impact of debris collection because in particular cases, young, inexperienced white stork females may lay more eggs compared to older individuals (Aguirre and Vergara 2007). What is more, clutch size is related to current food supply (Tortosa et al. 2003) and to conditions in the wintering grounds during the previous winter (Schamber et al. 2012; Tobolka et al. in review). Although numbers of collected debris may be an indicator of innovative behaviour (Borgia 1985) and the age-related experience (Sergio et al. 2011) of pair members, which may be reflected also in food provisioning, the influence of debris on developing nestlings is not equal. Several types of debris may produce negative consequences, e.g. plastic string which may cause entanglement (Antczak et al. 2010), rubber elements, plastic tape or string which may cause strangulation (Henry et al. 2011), or wire and other metal elements which may cause injuries. Along with the common occurrence of the incorporation of anthropogenic materials into nest structures, only a few cases of entanglement were noticed. During 8 years of studies, 0.73% of 2043 nestlings (from 728 nests) were found entangled (11 dead, 4 with fatal injuries necessitating euthanasia) and two cases of strangulation with plastic elements choked in nestling throats (Tobolka, unpublished data) during the ringing process. Hence, we recorded lethal consequences of collecting debris in only 2% of nests. However, the number of entangled nestlings may have been higher, as we did not detect mortality in earlier stages. In this study, nests were visited when chicks were old enough to ring, in age of 25–40 days. White stork nestlings spend ca 55 days in the nest, and their mortality varies from 21 to 85%, which is mainly due to varying weather conditions (Tobolka et al. 2015); entanglement may be another threat. This situation was also observed in the American crow in farmlands, where 5.6% of 195 nestlings were entangled in their nests (Townsend and Barker 2010). Environmental pollution with anthropogenic materials has been present only for the past several decades; therefore, incorporation of debris in nests by birds is a relatively new behaviour (Ptaszyk 1994). There are many reports of the incorporation of debris into nests by various bird species (e.g. marine colonial birds) (Votier et al. 2011; Verlis et al. 2014; Tavares et al. 2016), although the scale of this behaviour is still not known in detail. The white stork appears to be a species in which the phenomenon is currently developing, at least in Poland. It may be worth monitoring whether debris incorporation into nests becomes more widespread and exerts an impact on individuals and populations in future. However, the abundance of plastic string in the agricultural environment and its non-biodegradability (it only breaks into smaller and smaller fragments) make it necessary to constantly monitor the scale of entanglement and ingestion. Moreover, this behaviour may be widespread and may have an impact on more individuals/populations in the future (e.g. Antczak et al. 2010).

## Conclusions

Considering all aspects, the white stork adapts easily to anthropogenic changes and can use new nesting material available in the environment. In order to define the long-term consequences (i.e. costs of incorporating debris) and individual conditions for incorporating anthropogenic materials into nests, this behaviour should be continuously monitored, and more studies should be performed to understand this phenomenon better. Using more detailed methods (e.g. trapping cameras) will help us to identify the exact pattern of collecting debris for nests, as well as to determine which sex is primarily responsible for debris collection and when such incorporation takes place. Also, in this case, research on the impact of the age of individuals on this behaviour should be continued.

## Electronic supplementary material

ESM 1 The frequency of particular debris categories in the vicinity of white stork nests (DOCX 55 kb)

## References

[CR1] Aguirre JM, Vergara P (2007). Younger, weaker white stork (*Ciconia ciconia*) became the best breeders. Evol Ecol Res.

[CR2] Antczak M, Hromada M, Czechowski P, Tabor J, Zabłocki P, Grzybek J, Tryjanowski P (2010). A new material for old solutions—the case of plastic string used in great grey shrike nests. Acta Ethol.

[CR3] Avery-Gomm S, O’Hara PD, Kleine L, Bowes V, Wilson LK, Barry KL (2012). Northern fulmars as biological monitors of trends of plastic pollution in the eastern North Pacific. Mar Pollut Bull.

[CR4] Bairlein F, Perrins CM, Lebreton JD, Hirons GJM (1991). Population studies of white stork (*Ciconia ciconia*) in Europe. Bird population studies: relevance to conservation and management.

[CR5] Baker GB, Gales R, Hamilton S, Wilkinson V (2002). Albatrosses and petrels in Australia: a review of their conservation and management. Emu.

[CR6] Barbraud C, Barbraud JC (1999). Is there age assortative mating in the European white stork?. Waterbirds.

[CR7] Bates D, Maechler M, Bolker B, Walker S (2015). Fitting linear mixed-effects models using lme4. J Stat Softw.

[CR8] Blem CR, Blem LB, Harmata PJ (2002) Twine causes significant mortality in nestlings ospreys. Wilson Bull 114:528–529. https://doi.org/10.1676/00435643(2002)114[0528:TCSMIN]2.0.CO;2

[CR9] Bocheński M, Jerzak L (2006). Behaviour of the white stork (*Ciconia ciconia*): a review.

[CR10] Bond AL, Montevecchi WA, Guse N, Regular PM, Garthe S, Rail JF (2012). Prevalence and composition of fishing gear debris in the nests of northern gannets (*Morus bassanus*) are related to fishing effort. Mar Pollut Bull.

[CR11] Bond AL, Provencher JF, Elliot RD, Ryan PC, Rowe S, Jones IL, Robertson GJ, Wilhelm SI (2013). Ingestion of plastic marine debris by common and thick-billed murres in the northwestern Atlantic from 1985 to 2012. Mar Pollut Bullet.

[CR12] Borgia G (1985). Bowers quality, number of decorations and mating success of male satin bowerbirds (*Ptilonorhynchus violaceus*): an experimental analysis. Anim Behav.

[CR13] Canal D, Mulero-Pázmány M, Negro JJ, Sergio F (2016). Decoration increases the conspicuousness of raptor nests. PLoS One.

[CR14] Carney KM, Sydeman WJ (1999). A review of human disturbance effects on nesting colonial waterbirds. Waterbirds.

[CR15] Ciach M, Kruszyk R (2010). Foraging of white storks *Ciconia ciconia* on rubbish dumps on non-breeding grounds. Waterbirds.

[CR16] Coleman SE, Patricelli GA, Borgia G (2004). Variable females preferences drive complex male displays. Nature.

[CR17] Delignette-Muller ML, Dutang C (2015). Fitdistrplus: an R package for fitting distributions. J Stat Softw.

[CR18] Djerdali S, Tortosa FS, Hillstrom L, Doumandji S (2008). Food supply and external cues limit the clutch size and hatchability in the white stork *Ciconia ciconia*. Acta Ornithol.

[CR19] Djerdali S, Guerrero-Casado J, Tortosa FS (2016). The effects of colony size interacting with extra food supply on the breeding success of the white stork (*Ciconia ciconia*). J Ornithol.

[CR20] Dubiec A, Zagalska-Neubauer M (2006). Molecular techniques for sex identification in birds. Biol Lett.

[CR21] Eriksen M, Lebreton LCM, Carson HS, Thiel M, Moore CJ, Borerro JC, Galgani F, Ryan PG, Reisser J (2014). Plastic pollution in the world’s oceans: more than 5 trillion plastic pieces weighing over 250,000 tons afloat at sea. PLoS One.

[CR22] Fernandes M, Borges C, Simoes F, Caballero JM, Pacheco C, Franco C (2006). Molecular sexing of the black stork *Ciconia nigra*: sex ratios in the Portuguese population. Biota.

[CR23] Ferrer M, Penteriani V (2003). A process of pair formation leading to assortative mating: passive age-assortative mating by habitat heterogeneity. Anim Behav.

[CR24] Finkelstein ME, Doak DF, George D, Burnett J, Brandt J, Grantham J, Smith DR (2012). Lead poisoning and the deceptive recovery of the critically endangered California condor. Proc Natl Acad Sci.

[CR25] Flack A, Fiedler W, Blas J, Pokrovsky I, Kaatz M, Mitropolsky M, Aghababyan K, Fakriadis I, Makrigianni E, Jerzak L, Azafzaf H, Feltrup-Azafzaf C, Rotics S, Mokotjomela TM, Nathan R, Wikelski M (2016). Costs of migratory decisions: a comparison across eight white stork populations. Sci Adv.

[CR26] Gall SC, Thompson RC (2015). The impact of debris on marine life. Mar Pollut Bull.

[CR27] Gilbert NI, Correia RA, Silva JP, Pacheco C, Catry I, Atkinson PW, Gill JA, Franco AMA (2016). Are white storks addicted to junk food? Impacts of landfill use on the movement and behaviour of resident white storks (*Ciconia ciconia*) from a partially migratory population. Mov Ecol.

[CR28] Gregory MR (2009). Environmental implications of plastic debris in marine settings – entanglement, ingestion, smothering, hangers-on, hitch-hiking and alien invasions. Philos Trans R Soc B, Biol Sci.

[CR29] Hartwig E, Clemens T, Heckroth M (2007). Plastic debris as nesting material in a kittiwake (*Rissa tridactyla*) colony at the Jammerbugt, Northwest Denmark. Mar Pollut Bull.

[CR30] Henriksen K (2000). Man-made materials in nests of blackbirds. Dan Ornitol Foren Tidss.

[CR31] Henry PY, Wey G, Balança G (2011). Rubber band ingestion by a rubbish dump dweller, the white stork (*Ciconia ciconia*). Waterbirds.

[CR32] Houston DC, Mee A, McGrady M (2007). Why do condors and vultures eat junk?: the implications for conservation. J Raptor Res.

[CR33] Hoornweg D, Bhada-Tata P, Kennedy C (2013). Waste production must peak this century. Nature.

[CR34] Huin N, Croxall JP (1996). Fishing gear, oil and marine debris associated with seabirds at Bird Island, South Georgia, during 1993/1994. Mar Ornithol.

[CR35] Kruszyk R, Ciach M (2010). White storks, forage on rubbish dumps in Poland—a novel behaviour in population. Eur J Wildlife Res.

[CR36] Lee K, Jang YC, Hong S, Lee J, Kwon IK (2015). Plastic marine debris used as nesting materials of the endangered species black-faced spoonbill *Platalea minor* decreases by conservation activities. J Korean Soc Mar Environ Energy.

[CR37] Mee A, Rideout BA, Hamber JA, Todd JN, Austin G, Clark M, Wallace MP (2007). Junk ingestion and nestling mortality in a reintroduced population of California condors *Gymnogyps californianus*. Bird Conserv Int.

[CR38] Miranda AC, Murgui E, Hedblom M (2017). Mechanisms of behavioural change in urban animals: the role of microevolution and phenotypic plasticity. Ecology and conservation of birds in urban environments. Springer international publishing, Cham, Switzerland.

[CR39] Morin M, Conant S (1990). Nest substrate between native and introduced populations of Laysan finches. Wilson Bull.

[CR40] Ortega S, Sanchez-Macouzet O, Urrutia A, Rodriguez C, Drummond H (2017). Age-related parental care in a long-lived bird: implications for offspring development. Behav Ecol Sociobiol.

[CR41] Ożgo M, Bogucki Z (1999) Home range and intersexual differences in the foraging habitat use of a White Stork (*Ciconia ciconia*) breeding pair. In: Schulz H (ed). Weißstorchim Aufwind? - White Storks on the up? Proc. Int. Symp. White Stork Hamburg, 1996, NABU, (Nafurschutzbund Deutchland e, V), Bonn, pp 481–492

[CR42] Provencher JF, Bond AL, Avery-Gomm S, Borrelle SB, Bravo Rebolledo EL, Hammer S, Kuhn S, Lavers JL, Mallory ML, Trevail A, van Franeker JA (2017). Quantifying ingested debris in marine megafauna: a review and recommendations for standardization. Anal Methods.

[CR43] Ptaszyk J (1994). Binfadenaus PolypropylenalsUrsache des Todesjunger Weißstorche (*Ciconia ciconia*) und anderer Tiere. Prace Zakładu Biologii i Ekologii Ptaków UAM.

[CR44] Profus P (2006) Population changes and breeding ecology of the white stork *Ciconia ciconia* L. in Poland against a background of the European population. Synthesis. Stud Nat 50, Kraków

[CR45] Development Core Team R (2016). R: a language and environment for statistical computing.

[CR46] Radhamany D, Anoop Das KS, Abdul Azeez P, Sálim A, Wen L, Sreekala LK (2016). Usage of nest materials by house sparrow (*Passer domesticus*) along an urban to rural gradient in Coimbatore, India. Trop Life Sci Res.

[CR47] Ryan PG, Moore CJ, van Franeker JA, Moloney CL (2009). Monitoring the abundance of plastic debris in the marine environment. Philos Trans R Soc B, Biol Sci.

[CR48] Schamber JL, Sedinger JS, Ward DH (2012). Carry-over effects of winter location contribute to variation in timing of nest initiation and clutch size in black Brant (*Branta bernicla nigricans*). Auk.

[CR49] Schulz H, Cramp S, Simmons KEL (1998). *Ciconia ciconia* white stork. Birds of the western Palearctic, update 2.

[CR50] Seacor R, Ostovar K, Restani M (2014). Distribution and abundance of baling twine in the landscape near osprey (*Pandion haliaetus*) nests: implications for nestling entanglement. Can Field Nat.

[CR51] Sergio F, Blas J, Blanco G, Tanferna A, Lopez L, Lemus JA, Hiraldo F (2011). Raptor nest decorations are a reliable threat against conspecifics. Science.

[CR52] Slabbekoorn H, den Boer-Visser A (2006). Cities change the songs of birds. Curr Biol.

[CR53] Suárez-Rodríguez M, Lopez-Rull I, Macias Garcia C (2012). Incorporation of cigarette butts into nests reduces nest ectoparasite load in urban birds: new ingredients for an old recipe?. Biol Lett.

[CR54] Suárez-Rodríguez M, Macías Garcia C (2014). There is no such a thing as a free cigarette; lining nests with discarded butts brings short-term benefits, but causes toxic damage. J Evol Biol.

[CR55] Thompson RC, Moore CJ, Vom Saal FS, Swan SH (2009). Plastics, the environment and human health: current consensus and future trends. Philos Trans R Soc Lond Ser B Biol Sci.

[CR56] Tobolka M, Kuźniak S, Zolnierowicz KM, Sparks TH, Tryjanowski P (2013). New is not always better: low breeding success and different occupancy pattern in newly built nests of a long-lived species, the white stork *Ciconia ciconia*. Bird Study.

[CR57] Tobolka M, Zolnierowicz KM, Reeve NF (2015). The effect of extreme weather events on breeding parameters of the white stork (*Ciconia ciconia*). Bird Study.

[CR58] Tortosa FS, Caballero JM, Reyes-Lopez J (2002) Effect of rubbish dumps on breeding success in the white stork in southern Spain. Waterbirds 25:39–43. https://doi.org/10.1675/1524-4695(2002)025[0039:EORDOB]2.0.CO;2

[CR59] Tortosa FS, Pérez P, Hillström L (2003). Effect of food abundance on laying date and clutch size in the white stork *Ciconia ciconia*. Bird Study.

[CR60] Townsend AK, Barker CM (2014). Plastic and the nest entanglement of urban and agricultural crows. PLoS One.

[CR61] Tryjanowski P, Kosicki JZ, Kuźniak S, Sparks TH (2009). Long-term changes and breeding success in relation to nesting structures used by white stork (*Ciconia ciconia*). Ann Zool Fenn.

[CR62] Tryjanowski P, Sparks TH, Jerzak L (2006). The white stork in Poland: studies in biology, ecology and conservation.

[CR63] Vergara P, Gordo O, Aguirre JI (2010). Nest size, nest building behavior and breeding success in a species with nest reuse: the white stork *Ciconia ciconia*. Ann Zool Fenn.

[CR64] Verlis KM, Campbell ML, Wilson SP (2014) Marine debris is selected as nesting material by the brown booby (*Sula leucogaster*) within the Swain Reefs, Great Barrier Reef, Australia. Mar Pollut Bull 87:180–190. 10.1016/j.marpolbul.2014.07.060 10.1016/j.marpolbul.2014.07.060 25131418

[CR65] Votier SC, Archibald K, Morgan G, Morgan L (2011). The use of plastic debris as nesting material by a colonial seabird and associated entanglement mortality. Mar Pollut Bull.

[CR66] Wang Y, Chen S, Blair RB, Jiang P, Ding P (2009). Nest composition adjustments by Chinese bulbuls (*Pycnonotus sinensis*) in an urbanized landscape of Hangzhou (E China). Acta Ornithol.

[CR67] Wickham H (2009). ggplot2: elegant graphics for data analysis.

[CR68] Wilcox C, Van Sebille E, Hardesty BD (2015). Threat of plastic pollution to seabirds is global, pervasive, and increasing. Proc Natl Acad Sci.

[CR69] Young LC, Vanderlip C, Duffy DC, Afanasyev V, Shaffer SA (2009). Bringing home the trash: do colony-based differences in foraging distribution lead to increased plastic ingestion in Laysan albatrosses?. PLoS One.

