# Supplementary material for: Factors determining the occurrence of anthropogenic materials in nests of the white stork Ciconia ciconia
Source: Environ Sci Pollut Res Int. 2018 Mar 13;25(15):14726–33. doi: 10.1007/s11356-018-1626-x (PMC5973955; doi:10.1007/s11356-018-1626-x)
Supplement: Supplementary file 1 — The frequency of particular debris categories in the vicinity of white stork nests (DOCX 55 kb) [file 11356_2018_1626_MOESM1_ESM.docx]

SUPLEMENTARY MATERIALS

## Factors determining the occurrence of anthropogenic materials in nests of the White Stork *Ciconia ciconia*

Tab. S1 List of total debris found in White Storks nests.

| Debris | Kind of material | Standardized categories  (according to Provencher et al. 2017) | Number | Frequency (%) |
| --- | --- | --- | --- | --- |
| plastic strings | PLASTIC | THREADLIKE PLASTIC | 344 | 38.18 |
| foil | FOIL | SHEET PLASTIC | 295 | 32.74 |
| sponge | OTHER | OTHER | 65 | 7.21 |
| textile material | TEXTILE | OTHER | 63 | 6.99 |
| cardboard, paper | PAPER | OTHER | 41 | 4.55 |
| non woven crop cover | PLASTIC | SHEET PLASTIC | 13 | 1.44 |
| plastic pieces | PLASTIC | OTHER PLASTIC | 8 | 0.89 |
| plastic bag | PLASTIC | SHEET PLASTIC | 7 | 0.78 |
| potatoes net | PLASTIC | THREADLIKE PLASTIC | 7 | 0.78 |
| plastic tape | PLASTIC | OTHER PLASTIC | 6 | 0.67 |
| bale net | PLASTIC | THREADLIKE PLASTIC | 6 | 0.67 |
| work glove | TEXTILE | OTHER | 6 | 0.67 |
| polyurethane foam | OTHER | OTHER | 4 | 0.44 |
| tape | PLASTIC | OTHER PLASTIC | 4 | 0.44 |
| mineral wool | PLASTIC | OTHER PLASTIC | 3 | 0.33 |
| plywood | OTHER | OTHER | 3 | 0.33 |
| rubber glove | OTHER | OTHER | 3 | 0.33 |
| rubber | OTHER | OTHER | 2 | 0.22 |
| pet bottle | PLASTIC | OTHER PLASTIC | 2 | 0.22 |
| wet wipe | PLASTIC | PLASTIC | 2 | 0.22 |
| cigarette pack | PAPER | OTHER | 2 | 0.22 |
| material | TEXTILE | OTHER | 2 | 0.22 |
| cable/wire | OTHER | OTHER | 1 | 0.11 |
| panties | TEXTILE | OTHER | 1 | 0.11 |
| belt | TEXTILE | OTHER | 1 | 0.11 |
| plastic hanger | PLASTIC | OTHERPLASTIC | 1 | 0.11 |
| wire | OTHER | OTHER | 1 | 0.11 |
| wood | OTHER | OTHER | 1 | 0.11 |
| shoelace | TEXTILE | OTHER | 1 | 0.11 |
| pantyhose | TEXITLE | OTHER | 1 | 0.11 |
| fiberglass net | PLASTIC | THREADLIKE PLASTIC | 1 | 0.11 |
| tennis ball | OTHER | OTHER | 1 | 0.11 |
| shoe insole | TEXITLE | OTHER | 1 | 0.11 |
| aluminum foil | OTHER | OTHER | 1 | 0.11 |
| box of yogurt | PLASTIC | PLASTIC | 1 | 0.11 |
